# Healing through volunteering: psychological wellbeing in humanitarian missions

**DOI:** 10.3389/fpubh.2025.1729533

**Published:** 2025-12-02

**Authors:** Eman Hajr

**Affiliations:** Department of Otolaryngology–Head and Neck Surgery, Imam Mohammad Ibn Saud Islamic University (IMSIU), Riyadh, Saudi Arabia

**Keywords:** volunteering, cochlear implant, psychological wellbeing, medical team, mental health, mission

## Abstract

**Background:**

Psychological wellbeing is a critical component of overall health, particularly for healthcare professionals who face high-stress environments. Volunteering has been associated with positive mental health outcomes, yet limited research has explored its impact on specialized medical missions. This study investigates the psychological effects of participating in a cochlear implant humanitarian mission on healthcare professionals, assessing changes in their mental wellbeing before and after the experience.

**Methods:**

A descriptive, questionnaire-based study was conducted on volunteers who participated in a cochlear implant mission for Syrian children with hearing loss in Turkey. The Warwick-Edinburgh Mental Wellbeing Scale (WEMWBS) was administered in two phases—before and after the mission. A Generalized Estimating Equations (GEE) model was applied to assess changes in wellbeing scores, adjusting for socio-demographic predictors.

**Results:**

A total of 31 participants completed both phases. The average WEMWBS score significantly increased after the mission (*β* = 9.19, *p* < 0.001), indicating improved psychological wellbeing. Positive changes were observed across multiple wellbeing indicators, including optimism, confidence, and emotional resilience. Notably, participants with prior humanitarian experience showed greater wellbeing improvements. Despite the demanding nature of the mission, all participants expressed high satisfaction, with 100% willing to participate in future missions.

**Conclusion:**

Participation in a cochlear implant humanitarian mission positively influenced healthcare professionals’ psychological wellbeing, reinforcing the value of structured volunteer programs in mitigating stress and enhancing resilience. These findings highlight the potential role of medical volunteering in improving mental health and professional fulfilment among healthcare workers.

## Introduction

Psychological wellbeing is a key component of overall health and essential for effective functioning in demanding professions such as healthcare. Medical professionals work under continuous emotional and physical pressure, with long hours, critical decision-making, and exposure to distressing situations contributing to stress, anxiety, and burnout (1). Even before entering practice, medical and nursing students experience substantial psychological strain, with higher rates of depression, anxiety, and emotional exhaustion than students in other academic fields (2, 3). Ensuring psychological resilience early in training is therefore crucial for long-term personal and professional functioning.

The mental health burden on healthcare workers became even more evident during the COVID-19 pandemic, when high levels of anxiety, depression, insomnia, and post-traumatic stress were widely reported (4–6). This has reinforced the need for strategies that support the psychological wellbeing of healthcare professionals at all stages of their careers.

Volunteering has been recognized as an activity that promotes mental and emotional wellbeing by enhancing meaning, life satisfaction, and emotional resilience (7–12). Participation in voluntary work is associated with reduced depressive symptoms, greater sense of purpose, improved coping abilities, and strengthened social connectedness. Recent studies further confirm that volunteering contributes positively to psychological and social wellbeing across different age groups, improving mood, enhancing social relationships, and supporting healthier lifestyles (13–16).

Volunteering in specialized medical missions, such as high-intensity cochlear implant campaigns, involves unique professional and emotional demands—including rapid clinical decision-making, coordinated teamwork, and delivering life-changing interventions to vulnerable populations. These environments combine clinical pressure with profound personal fulfilment, which may enhance psychological wellbeing through a strong sense of purpose, teamwork cohesion, and immediate visible patient impact. Despite extensive evidence linking volunteering to mental health benefits, the psychological effects of participation in highly specialized medical humanitarian missions remain underexplored.

This study aims to address this gap by examining the psychological wellbeing of healthcare professionals involved in a large-scale cochlear implant volunteer program. Understanding these effects may help guide future initiatives that leverage structured volunteering as a strategy to support the mental health of medical teams.

## Methods

### Study design and operational context

This study employed a descriptive pre–post design to evaluate the experiences of healthcare volunteers during a large-scale humanitarian cochlear implant (CI) mission conducted in Turkey. The mission was organized with support from the Saudi government and involved a multidisciplinary team operating across five surgical theatres. Over a span of three consecutive days, a total of 142 cochlear implant surgeries were performed. The study included 31 healthcare volunteers representing various specialties integral to cochlear implant procedures. These disciplines encompassed:

- Surgeons
- Audiologists
- Nurses
- Speech-language pathologists
- Anesthetists
- Anesthesia technicians
- Biomedical engineers
- Coordinators
- Additional support personnel

All participating volunteers possessed prior clinical experience in cochlear implant programs within Saudi Arabia and volunteered voluntarily for participation in the mission. Documenting these demographic and professional variables enhances the clarity of the study design, ensuring transparency and facilitating comprehensive analysis aligned with peer review standards.

### Instrument and validity

Psychological wellbeing was assessed using the *Warwick–Edinburgh Mental Wellbeing Scale (WEMWBS)*, a validated and widely used instrument in mental health research. The tool demonstrates excellent psychometric properties, including:

- *Internal consistency*: Cronbach’s *α* = 0.89–0.93
- *Test–retest reliability*: 0.83–0.91
- *Established construct validity* across diverse populations, including healthcare providers

These properties support its suitability for evaluating positive mental wellbeing in this cohort and address the reviewer’s request for clarification on instrument validity.

### Data collection procedures

Data were collected in two phases:

1) *Pre-mission phase:*
  Before traveling, participants attended an online briefing session during which the study purpose and procedures were explained. They then completed the baseline WEMWBS questionnaire electronically.
2) *Post-mission phase:*
  Upon returning to Saudi Arabia and completing the surgical mission, participants received the follow-up questionnaire containing the same WEMWBS items, along with additional questions assessing mission satisfaction and willingness for future participation.

To maintain confidentiality while enabling paired analysis, participants generated a unique personal code rather than providing identifiable data.

The decision to focus on this specific volunteer cohort was based on their shared exposure to identical clinical, emotional, and environmental demands during the mission. This homogeneity ensured accurate pre–post assessment of wellbeing changes attributable to the humanitarian experience. Access to volunteers outside the mission was neither feasible nor aligned with the study objective, which was to assess psychological effects within the context of a high-intensity CI humanitarian program.

### Statistical analysis

All WEMWBS items were scored using a 5-point Likert scale (1 = none of the time, 5 = all the time), producing total scores ranging from 14 to 70. Descriptive statistics were used for demographic variables. A *Generalized Estimating Equations (GEE)* model was applied to evaluate changes in WEMWBS scores before and after the mission while adjusting for age, gender, profession, and number of prior humanitarian experiences.

### Findings

Thirty-one participants completely responded to the two phases of the questionnaire with an average age of 36.2 ± 8.3 years, among whom, males represented 58.1 and 41.9% were females. About 25.8% of participants were surgeons, audiologists and nurses were equally represented 16.1% for each, 9.7% were speech pathologists, and the remaining 10 participants represented about 32.3% were equally distributed between anesthetists, anesthesia technicians, biomedical engineers, coordinators, and others.

About 45.2% of participants took part in a humanitarian mission for cochlear implantation for the first time, 32.3% participated once before, and for 16.1% of participants it was the third time, while for 6.5% of participants it was the fourth time or more as described in Table 1.

**Table 1:** Descriptive analysis for participants socio-demographic characteristics:

Socio-demographics	Variable	Overall (*N* = 31)
Age (years)	Mean (SD)	36.2 (8.3)
Gender	Female	13 (41.9)
	Male	18 (58.1)
Role	Anesthesia technician	2 (6.5)
	Anesthetist	2 (6.5)
	Audiologist	5 (16.1)
	Biomedical Engineer	2 (6.5)
	Coordinator	2 (6.5)
	Speech pathologist	3 (9.7)
	Surgeon	8 (25.8)
	Nurse	5 (16.1)
	Other	2 (6.5)
Number of participations	This is my first time	14 (45.2)
	This is my second time	10 (32.3)
	This is my third time	5 (16.1)
	This is my fourth or more	2 (6.5)

### Warwick-Edinburgh mental wellbeing scale responses

Table 2 illustrates that before starting the humanitarian mission, about 74.0% of participants, all the time and often, were feeling optimistic about the future which increased to approximately 97.0% after finishing the mission. Also, about 77.0% were feeling useful, all the time and often, before the mission that changed to 100% after finishing it. While only 32.0% of participants were feeling relaxed all the time and often before the mission that increased to 68.0% approximately after it. Furthermore, about 68.0% of participants were interested in other people, all the time and often, before the mission, while increased after it to 90.0% approximately. And about 55.0% were having energy to spare, all the time and often, before starting the humanitarian mission that increased to 90.0% after finishing the mission.

**Table 2:** Descriptive analysis for Warwick-Edinburgh mental wellbeing scale (WEMWBS) questionnaire items before vs. after the humanitarian mission:

WEMWBS items	Responses	Before mission	After mission
I’ve been feeling optimistic about the future.	None of the time	0 (0.0)	0 (0.0)
	Rarely	2 (6.5)	0 (0.0)
	Sometimes	6 (19.4)	1 (3.2)
	Often	11 (35.5)	11 (35.5)
	All of the time	12 (38.7)	19 (61.3)
I’ve been feeling useful.	None of the time	1 (3.2)	0 (0.0)
	Rarely	2 (6.5)	0 (0.0)
	Sometimes	4 (12.9)	0 (0.0)
	Often	6 (19.4)	7 (22.6)
	All of the time	18 (58.1)	24 (77.4)
I’ve been feeling relaxed.	None of the time	4 (12.9)	1 (3.2)
	Rarely	6 (19.4)	0 (0.0)
	Sometimes	11 (35.5)	9 (29.0)
	Often	4 (12.9)	8 (25.8)
	All of the time	6 (19.4)	13 (41.9)
I’ve been feeling interested in other people	None of the time	1 (3.2)	0 (0.0)
	Rarely	2 (6.5)	0 (0.0)
	Sometimes	7 (22.6)	3 (9.7)
	Often	10 (32.3)	7 (22.6)
	All of the time	11 (35.5)	21 (67.7)
I’ve had energy to spare.	None of the time	2 (6.5)	0 (0.0)
	Rarely	4 (12.9)	3 (9.7)
	Sometimes	8 (25.8)	0 (0.0)
	Often	8 (25.8)	10 (32.3)
	All of the time	9 (29.0)	18 (58.1)
I’ve been dealing with problems well.	None of the time	0 (0.0)	0 (0.0)
	Rarely	2 (6.5)	0 (0.0)
	Sometimes	8 (25.8)	1 (3.2)
	Often	9 (29.0)	11 (35.5)
	All of the time	12 (38.7)	19 (61.3)
I’ve been thinking clearly.	None of the time	0 (0.0)	0 (0.0)
	Rarely	4 (12.9)	0 (0.0)
	Sometimes	6 (19.4)	3 (9.7)
	Often	9 (29.0)	6 (19.4)
	All of the time	12 (38.7)	22 (71.0)
I’ve been feeling good about myself.	None of the time	1 (3.2)	0 (0.0)
	Rarely	3 (9.7)	0 (0.0)
	Sometimes	6 (19.4)	3 (9.7)
	Often	9 (29.0)	5 (16.1)
	All of the time	12 (38.7)	23 (74.2)
I’ve been feeling close to other people.	None of the time	1 (3.2)	0 (0.0)
	Rarely	2 (6.5)	0 (0.0)
	Sometimes	8 (25.8)	4 (12.9)
	Often	11 (35.5)	7 (22.6)
	All of the time	9 (29.0)	20 (64.5)
I’ve been feeling confident.	None of the time	0 (0.0)	0 (0.0)
	Rarely	3 (9.7)	1 (3.2)
	Sometimes	8 (25.8)	2 (6.5)
	Often	7 (22.6)	4 (12.9)
	All of the time	13 (41.9)	24 (77.4)
I’ve been able to make up my own mind about things.	None of the time	0 (0.0)	0 (0.0)
	Rarely	3 (9.7)	1 (3.2)
	Sometimes	1 (3.2)	1 (3.2)
	Often	12 (38.7)	6 (19.4)
	All of the time	15 (48.4)	23 (74.2)
I’ve been feeling loved.	None of the time	0 (0.0)	0 (0.0)
	Rarely	3 (9.7)	1 (3.2)
	Sometimes	5 (16.1)	2 (6.5)
	Often	11 (35.5)	4 (12.9)
	All of the time	12 (38.7)	24 (77.4)
I’ve been interested in new things.	None of the time	0 (0.0)	0 (0.0)
	Rarely	3 (9.7)	0 (0.0)
	Sometimes	6 (19.4)	1 (3.2)
	Often	7 (22.6)	7 (22.6)
	All of the time	15 (48.4)	23 (74.2)
I’ve been feeling cheerful.	None of the time	0 (0.0)	0 (0.0)
	Rarely	2 (6.5)	0 (0.0)
	Sometimes	8 (25.8)	2 (6.5)
	Often	6 (19.4)	3 (9.7)
	All of the time	15 (48.4)	26 (83.9)

Also, about 68.0% were dealing with problems well, all of the time and often, before starting the mission that changed to approximately 97.0% after it. And about 68.0% believed that they were thinking clearly, all the time and often before the mission that increased to approximately 90.0% after finishing it. Also, about 68.0% felt good about themselves, all the time and often, before starting the mission that increased to approximately 90.0% after it.

**Figure 1:**
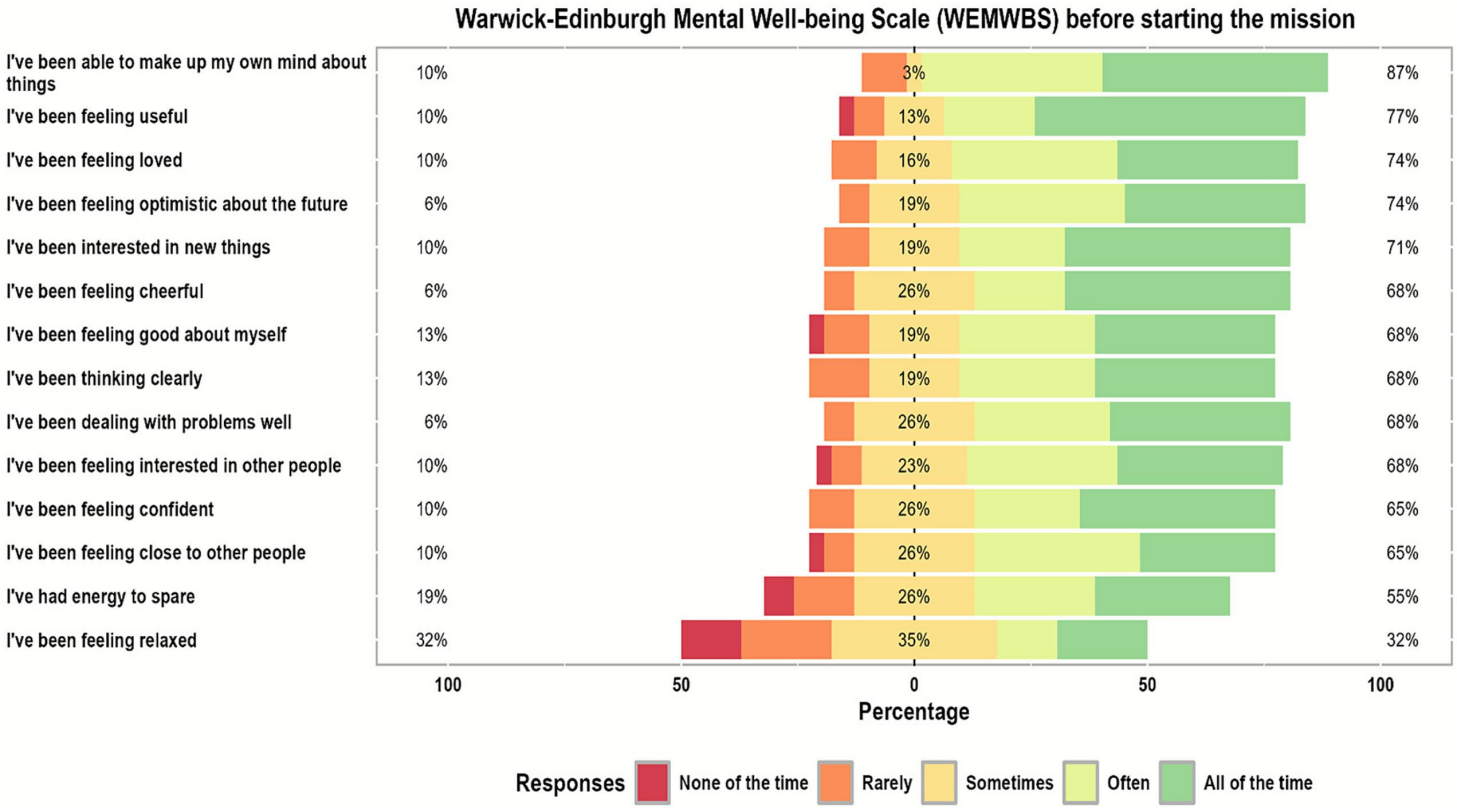
Likert scale responses for Warwick-Edinburgh mental wellbeing scale (WEMWBS) questionnaire items of the study participants before starting the mission.

**Figure 2:**
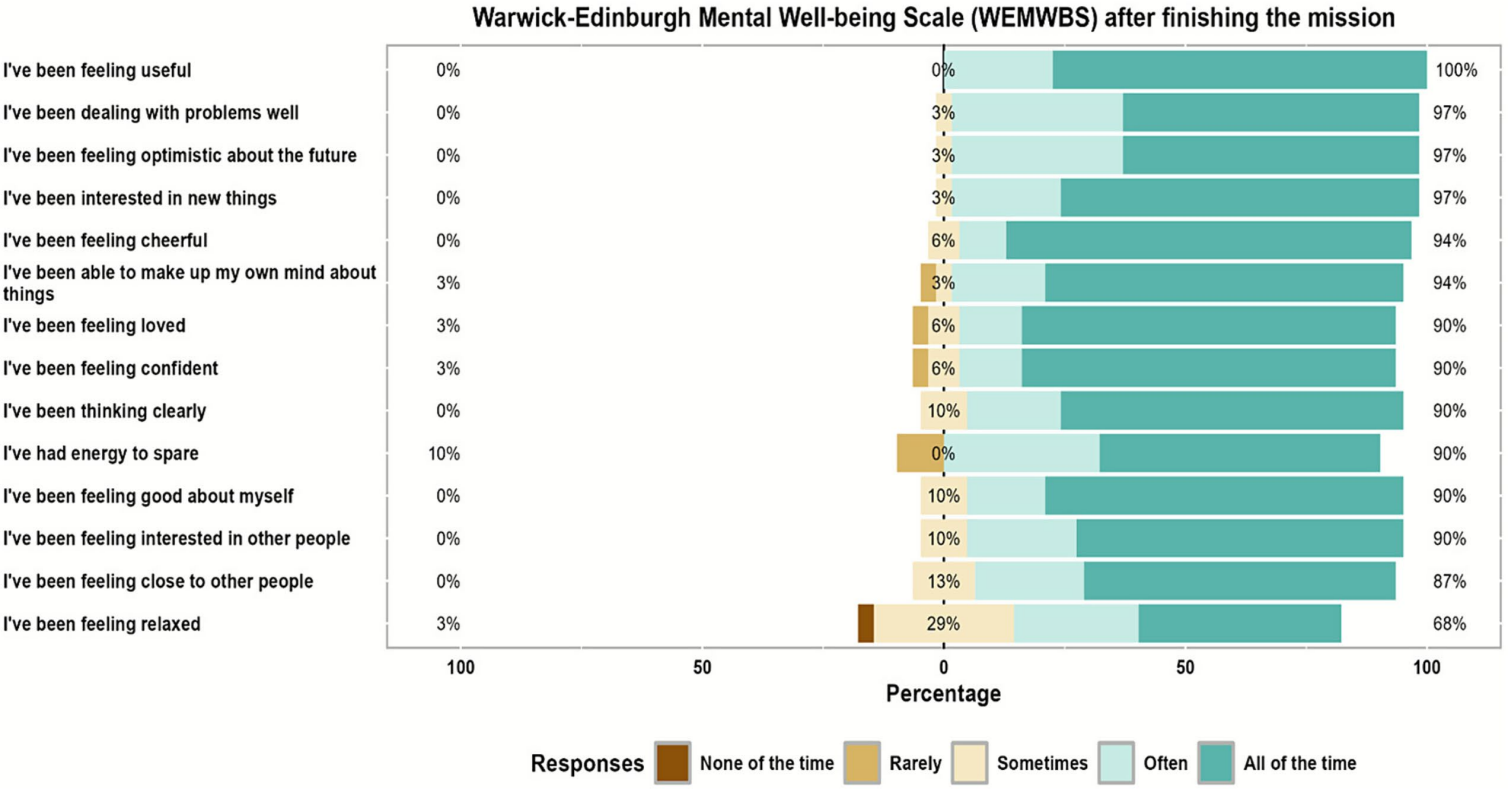
Likert scale responses for Warwick-Edinburgh mental wellbeing scale (WEMWBS) questionnaire items of the study participants after finishing the mission.

**Figure 3:**
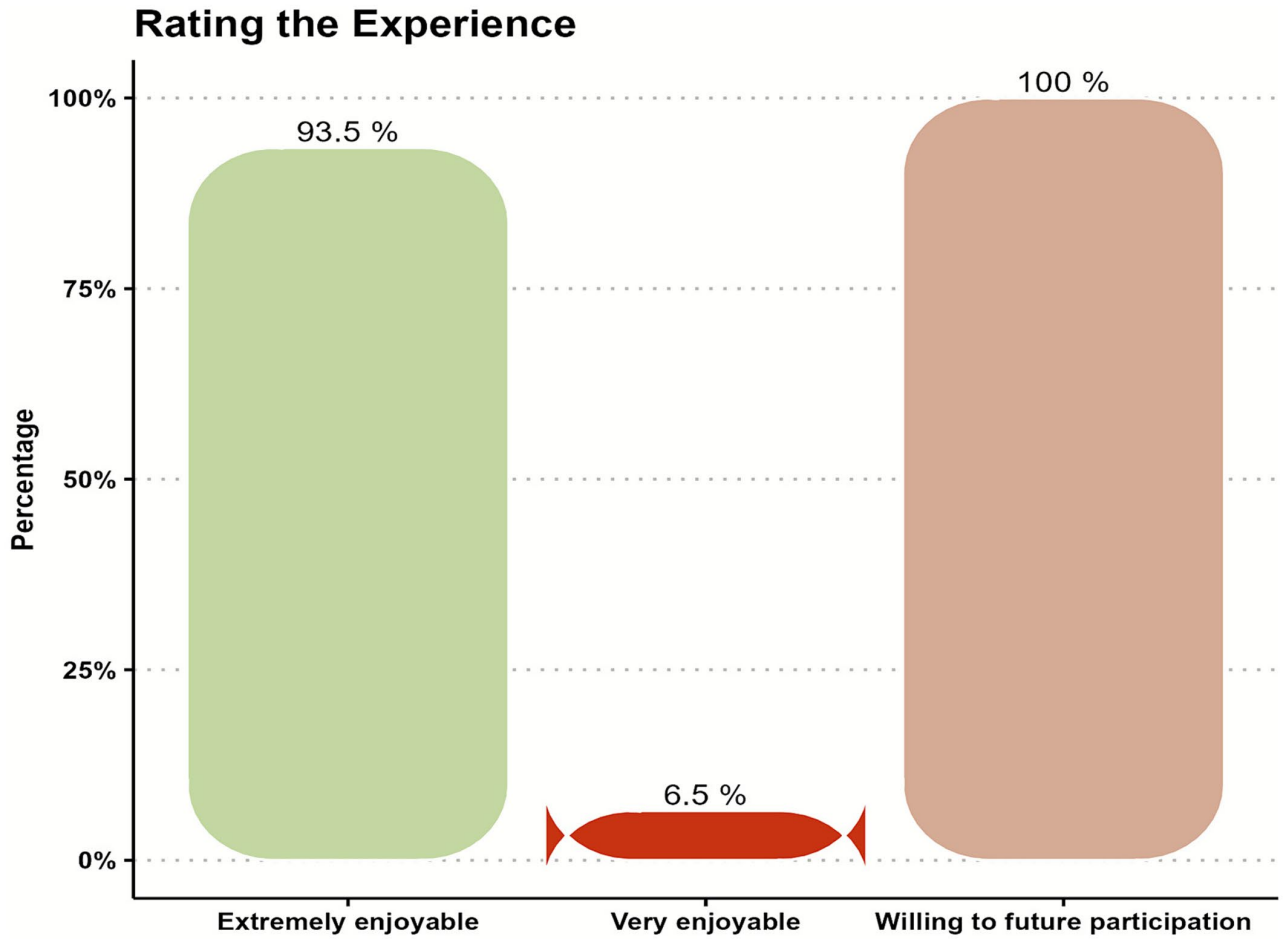
Rating the overall enjoyment of the humanitarian experience among study participants.

Furthermore, about 65.0% of participants felt close to other people, all the time and often, before the mission which increased to approximately 87.0% after finishing it. Also, about 65.0% were feeling confident, all the time and often, before the mission and increased to approximately 90.0% after finishing it. While, about 87.0% of participants believed that they were able to make up their minds about things, all the time and often, before starting the mission that increased to approximately 94.0% after finishing it. Also, about 74.0% were feeling loved, all the time and often, before the mission that changed to 90.0% approximately after it. Moreover, 71.0% of participants were interested in new things, all the time and often, before starting the mission that increased to approximately 97.0% after finishing it. While about 68.0% of participants were feeling cheerful, all the time and often, before starting the humanitarian mission that increased to 94.0% approximately after finishing it (see Figures 1, 2). After finishing the mission all participants were asked to rate their experience where 93.5% of them felt extremely enjoyable and the remaining 6.5% were very enjoyable and 100% of them were willing to future participation in such humanitarian missions as shown in Figure 3.

### Warwick-Edinburgh mental wellbeing scale total score

**Figure 4:**
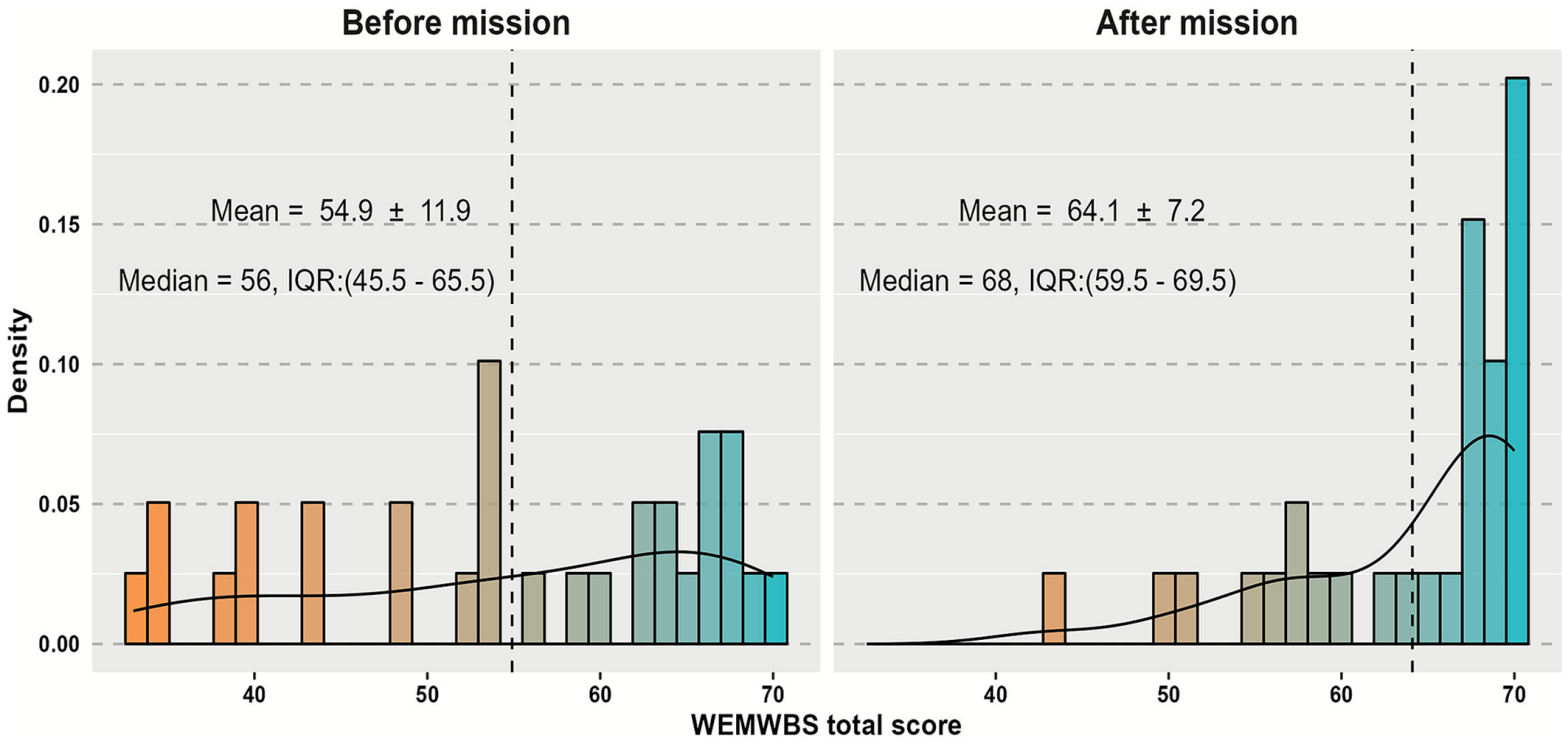
Warwick-Edinburgh mental wellbeing scale (WEMWBS) questionnaire total score before vs. after the humanitarian mission; (IQR: Interquartile range).

Figure 4 demonstrated the total score resulting from summing the score of the 14 questionnaire items in both phases showing that the average score of participants before starting the mission was 54.9 ± 11.9 having a median of 56 with interquartile range (IQR) from 45.5 to 65.5, while the average total score increased after finishing the mission to 64.1 ± 7.2 with a median of 68 and IQR from 59.5 to 69.5.

### The change in the WEMWBS mean score after the humanitarian mission in Table 3

**Table 3:** Generalized Estimating Equations (GEE) model predicting the change in the WEMWBS standardized mean score after the humanitarian mission adjusting for the potential socio-demographic predictors.

Predictors	WEMWBS total score		
	Estimates (*β*)	95% CI ^1^	*p*-value
Before mission	Reference group		
After mission	9.19	4.80–13.59	<0.001***
Age	−0.32	−0.91 to 0.28	0.301
Female	Reference group		
Male	4.41	−2.38 to 11.20	0.203
Anesthesia technician	Reference group		
Anesthetist	−7.01	−17.98 to 3.97	0.211
Audiologist	−10.71	−20.59 to −0.83	0.034*
Biomedical engineer	−9.26	−19.05 to 0.53	0.064
Coordinator	−6.31	−17.85 to 5.22	0.283
Speech pathologist	−8.75	−18.26 to 0.75	0.071
Surgeon	−10.57	−17.56 to −3.57	0.003**
Nurse	−8.63	−17.16 to −0.10	0.047*
Other	−0.36	−7.77 to 7.05	0.924
This is my first time	Reference group		
This is my second time	1.81	−4.41 to 8.02	0.569
This is my third time	7.33	−3.09 to 17.75	0.168
This is my fourth or more	11.63	1.62–21.63	0.023*

^1^95% CI: 95% confidence interval. *0.05 ≥ *p* ≥ 0.01, **0.01 > *p* ≥ 0.001, and ***p < 0.001.

Bold values indicate statistically significant results (*p* < 0.05).

The Generalized Estimating Equations (GEE) model showed that the mean score increased significantly among participants after finishing the mission by approximately 9.2 points compared to before it (*β* = 9.19, with 95%CI: (4.80–13.59), *p* < 0.001) adjusting for participants’ age, gender, role of participation, and the numbers of participations. While being an audiologist, a surgeon, and a nurse associated with statistically significant decreased scores by about 10.7 points, 10.6 points, and 8.6 points, respectively if compared to the anesthesia technicians (*β* = −10.71, with 95%CI: (−20.59 to −0.83), *p* = 0.034), (*β* = −10.57, with 95%CI: (−17.56 to −3.57), *p* = 0.003), and (*β* = −8.63, with 95%CI: (−17.16 to −0.10), *p* = 0.047), respectively.

Also, the biomedical engineers and speech pathologists showed observed decreased scores by about 9.3 points and 8.8 points, respectively, but non-statistically significant, if compared to the anesthesia technicians as well (*β* = −9.26, with 95%CI: (−19.05 to 0.53), *p* = 0.064) & (*β* = −8.75, with 95%CI: (−18.26 to 0.75), *p* = 0.071), respectively.

**Figure 5:**
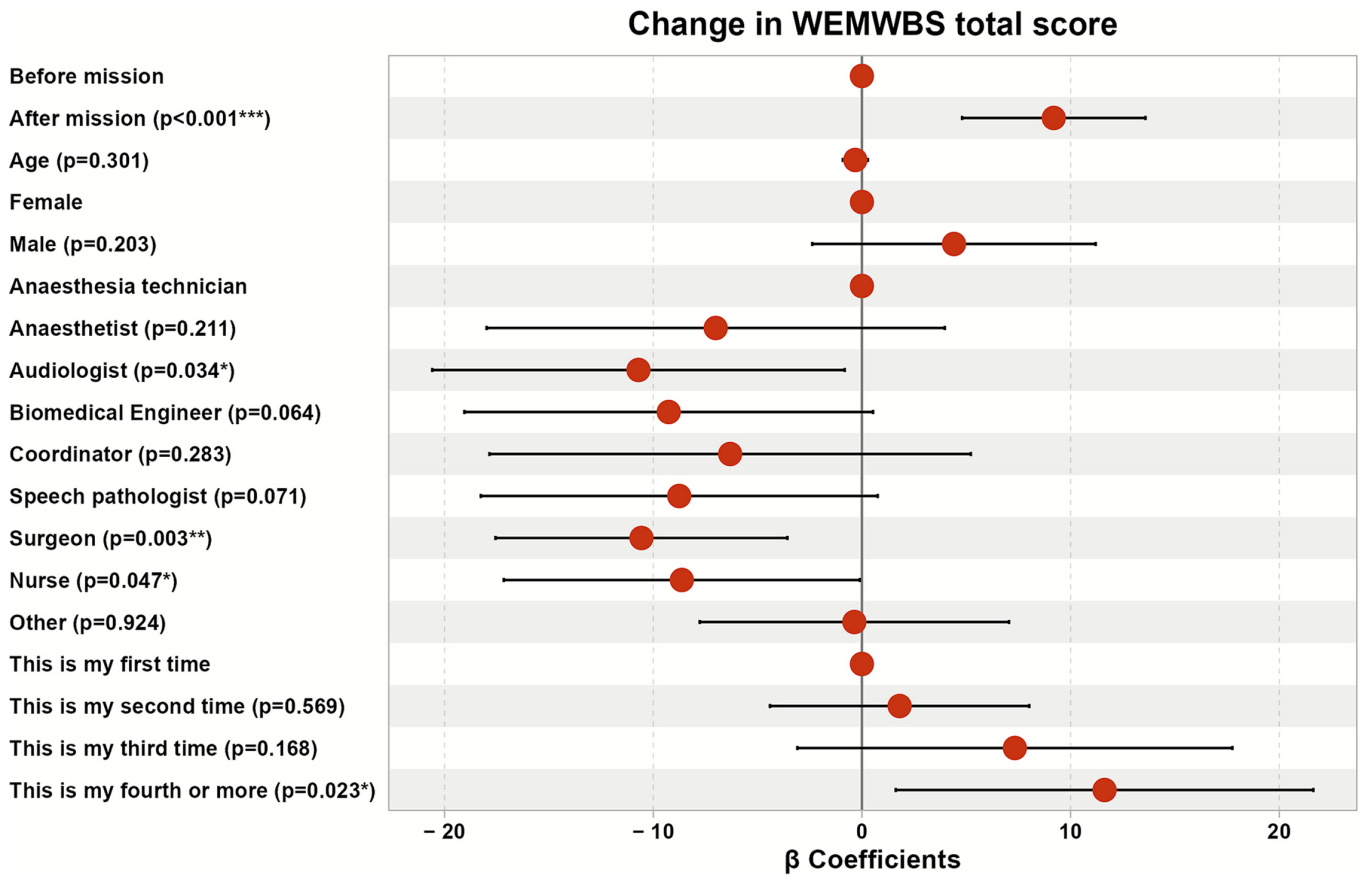
Forest plot predicting the change in the WEMWBS total score after the humanitarian mission adjusting for the potential socio-demographic predictors.

Furthermore, the study participants whose participation was the fourth one or more showed a statistically significant increase in the total score of about 11.6 points compared to those participating for the first time (*β* = 11.63, with 95%CI: (1.62–21.63), *p* = 0.023). (see the forest plot in Figure 5).

## Discussion

This study provides an important perspective on the psychological benefits of medical volunteering in a highly demanding humanitarian context. The cochlear implant campaign examined in this study represents a uniquely intense form of humanitarian medical work, characterized by highly specialized surgical tasks, tight time constraints, and complex team coordination. Despite these pressures, participation in the mission was associated with a substantial improvement in overall psychological wellbeing as reflected by a significant increase in total WEMWBS scores (*β* = 9.19, *p* < 0.001). This aligns with evidence that volunteering—particularly when it involves meaningful, high-impact service—enhances emotional resilience, purpose, and psychological functioning (8, 11, 13, 16).

Beyond the overall improvement, item-level WEMWBS analysis revealed notable gains in optimism, sense of usefulness, confidence, social connectedness, energy, and cognitive clarity. These findings mirror patterns described in recent volunteerism literature, which demonstrates that engaging in altruistic service promotes positive affect, increases perceived purpose, and strengthens social bonds, even in high-stress environments (16, 17). Importantly, the increase in feelings of closeness to others underscores the role of teamwork in humanitarian missions—cochlear implant campaigns require close interprofessional coordination between surgeons, audiologists, anesthesia technicians, speech therapists, and engineers, fostering a shared sense of achievement and belonging.

However, the magnitude of psychological improvement varied by professional role. Anesthesia technicians experienced the greatest increase in wellbeing, whereas surgeons, audiologists, and nurses demonstrated smaller or changes in comparison. This likely reflects role-specific pressures: surgeons and audiologists manage highly specialized and cognitively demanding tasks under significant time pressure, including surgical decision-making, intraoperative precision, perioperative assessments, and postoperative fitting responsibilities. Similar role-based disparities have been reported in other humanitarian contexts, where frontline or high-stakes roles experience higher stress despite the benefits of volunteering (18).

Prior humanitarian experience emerged as an important moderator. Volunteers who had participated in four or more missions showed significantly greater improvements in psychological wellbeing than first-time volunteers. This cumulative effect aligns with evidence that repeated volunteering builds emotional resilience and reinforces coping mechanisms over time (12). Familiarity with mission logistics, confidence in one’s professional role, and realistic expectations may all contribute to this enhanced psychological benefit.

Overall, these findings support a growing body of literature demonstrating that volunteering is associated with improved mental wellbeing, enhanced sense of meaning, and increased social connectedness (7, 8, 11, 16). The unique demands of large-scale cochlear implant missions—rapid surgical throughput, interprofessional collaboration, high emotional reward, and direct patient impact—may amplify these benefits.

This study also highlights the importance of structured, well-organized volunteer programs as a strategy to support the psychological health of healthcare professionals. Such initiatives may act as protective experiences against burnout, offering opportunities for meaningful contribution, teamwork, and renewed professional purpose.

Nevertheless, the study has limitations. The sample size was small, and long-term follow-up was not conducted, preventing conclusions about the durability of observed improvements. Additionally, although the mission setting uniquely represents high-intensity medical volunteering, comparisons with other forms of clinical and non-clinical volunteer work were not explored. Future research should examine long-term psychological trajectories, compare different volunteering models, and explore how role-specific stressors influence psychological outcomes across medical professions.

## Conclusion

This study demonstrates that participation in a high-impact humanitarian cochlear implant mission significantly enhances psychological wellbeing, with improvements observed across multiple emotional, cognitive, and social wellbeing indicators. The findings suggest that structured medical volunteering programs not only benefit recipients but also serve as a valuable tool for enhancing resilience among healthcare professionals. Future research should continue to explore the long-term effects of such initiatives and identify ways to maximize their psychological benefits for all participants.

## Data Availability

The raw data supporting the conclusions of this article will be made available by the authors, without undue reservation.
